# Design and validation of a disease network of inflammatory processes in the NSG-UC mouse model

**DOI:** 10.1186/s12967-017-1368-4

**Published:** 2017-12-28

**Authors:** Henrika Jodeleit, Pia Palamides, Florian Beigel, Thomas Mueller, Eckhard Wolf, Matthias Siebeck, Roswitha Gropp

**Affiliations:** 1 Institute of Molecular Animal Breeding and Biotechnology and Laboratory for Functional Genome Analysis (LAFUGA), Gene Center, LMU Munich, 81377 Munich, Germany; 2 Department of General- Visceral-, and Transplantation Surgery, Hospital of the University of Munich, Nussbaumstr. 20, 80336 Munich, Germany; 3 Department of Medicine II-Grosshadern, Ludwig-Maximilians-University (LMU), Marchioninistr. 15, 81377 Munich, Germany; 4 Julius von Sachs Institute, University of Würzburg, 97082 Würzburg, Germany; 5 Present Address: Department of Medicinal Microbiology, Max von Pettenkofer Institute, 80336 Munich, Germany

**Keywords:** Ulcerative colitis, Disease network, Autoimmunity, Inflammatory bowel disease, NSG, NSG-UC

## Abstract

**Background:**

Ulcerative colitis (UC) is a highly progressive inflammatory disease that requires the interaction of epithelial, immune, endothelial and muscle cells and fibroblasts. Previous studies suggested two inflammatory conditions in UC-patients: ‘acute’ and ‘remodeling’ and that the design of a disease network might improve the understanding of the inflammatory processes. The objective of the study was to design and validate a disease network in the NOD-SCID IL2rγ^null^ (NSG)-UC mouse model to get a better understanding of the inflammatory processes.

**Methods:**

Leukocytes were isolated from the spleen of NSG-UC mice and subjected to flow cytometric analysis. RT-PCR and RNAseq analysis were performed from distal parts of the colon. Based on these analyses and the effects of interleukins, chemokines and growth factors described in the literature, a disease network was designed. To validate the disease network the effect of infliximab and pitrakinra was tested in the NSG-UC model. A clinical- and histological score, frequencies of human leukocytes isolated from spleen and mRNA expression levels from distal parts of the colon were determined.

**Results:**

Analysis of leukocytes isolated from the spleen of challenged NSG-UC mice corroborated CD64, CD163 and CD1a expressing CD14+ monocytes, CD1a expressing CD11b+ macrophages and HGF, TARC, IFNγ and TGFß1 mRNA as inflammatory markers. The disease network suggested that a proinflammatory condition elicited by IL-17c and lipids and relayed by cytotoxic T-cells, Th17 cells and CD1a expressing macrophages and monocytes. Conversely, the remodeling condition was evoked by IL-34 and TARC and promoted by Th2 cells and M2 monocytes. Mice benefitted from treatment with infliximab as indicated by the histological- and clinical score. As predicted by the disease network infliximab reduced the proinflammatory response by suppressing M1 monocytes and CD1a expressing monocytes and macrophages and decreased levels of IFNγ, TARC and HGF mRNA. As predicted by the disease network inflammation aggravated in the presence of pitrakinra as indicated by the clinical and histological score, elevated frequencies of CD1a expressing macrophages and TNFα and IFNγ mRNA levels.

**Conclusions:**

The combination of the disease network and the NSG-UC animal model might be developed into a powerful tool to predict efficacy or in-efficacy and potential mechanistic side effects.

**Electronic supplementary material:**

The online version of this article (10.1186/s12967-017-1368-4) contains supplementary material, which is available to authorized users.

## Background

Inflammation in ulcerative colitis (UC) is a highly dynamic, spatial and temporal response and involves the communication of epithelial-, immune-, endothelial- and muscle cells and fibroblasts [1]. In contrast to Crohn’s disease, UC is considered a Th2 characterized inflammation. However, the role of Th2 cytokines has not been conclusively demonstrated [2, 3]. To get a better understanding of the processes in UC, we took on a different approach and considered the Th2 arm as the driver of wound healing ultimately leading to remodeling of the colon architecture [1, 4]. Based on the assumption that epithelial cells play an active role in the inflammatory process by releasing cytokines to direct immune responses we designed a panel of immune cells, cytokines and growth factors to develop immune profiles of UC patients. This profiling led to the identification of two inflammatory conditions referred to as ‘acute’ and ‘remodeling’, the latter of which was favored by treatment with infliximab [4]. The acute inflammatory condition was characterized by immune cells supporting the adaptive immunity, thymus and activation regulated chemokine (TARC) and hepatic growth factor (HGF). In contrast, the remodeling condition was characterized by innate immune cells such as NK T cells and TSLPR expressing CD14+ monocytes, periostin and transforming growth factor (TGF) ß1. The observation of an acute inflammatory arm was corroborated by data showing elevated frequencies of CD8+-, Th1- and Th17-T-cells in UC patients with active disease [5]. Furthermore, immune profiling of UC patients led to the identification of CD1a expressing CD11b+ macrophages and CD14+ monocytes as biological markers for UC [4]. These cells have been shown to present self-lipids to conventional αß CD4+ T cells to evoke an autoimmune response [6–8]. Finally, analysis of autoantibody levels suggested that autoimmunity might play a crucial role in a subgroup of patients (our own results). Thus, there seems to be a strong proinflammatory response which might be driven by IL-17 secreted by Th17 cells and by Th1 cells arising from Th17 cells in the presence of IL-6 [9, 10] or Th1 cells activated via CD1a providing an explanation for the responsiveness to TNFα blockers of a subgroup of UC patients. All these observations suggested that a map of the disease network illustrating the interaction and communication of immune-, epithelial cells and fibroblast may be a promising approach to understand inflammatory processes in UC and to predict efficacy and potential side effects of therapeutics. For the design and validation of the disease network, we took advantage of a mouse model of UC that is based on NOD-SCID IL2rγ^null^ (NSG) mice reconstituted with PBMC from human patients with UC (NSG-UC) [11]. Most of the cell types identified as crucial in UC patients could also be detected in spleen or colon of challenged mice. As in our previous hypothesis [1], the disease network was based on the assumption that damaged epithelial cells release signals to direct immune responses. However, we expanded the view and included IL-7, IL-17c, and lipids as factors to drive inflammation in UC. IL-7 is secreted by intestinal epithelial- and goblet cells to activate and to induce proliferation of T-cells and thus acts probably as an amplifier of a given immunological direction [12]. IL-17c promotes IL-17 responses of Th17 cells and autoimmunity [13]. IL-17A that is released by Th17 cells acts on epithelial cells to induce the release of IL-8 and CXCL1 to attract neutrophils and induce mucus production [14]. Both, IL-17A and IL-17c act on fibroblasts to release IL-6 [14]. In the presence of IL-6 and other factors, Treg can adopt a Th17 phenotype thus skipping the inflammatory balance twofold: by diminishing the Treg and by increasing the Th17 pool [15].

IL-33 has been described as an activator of innate lymphoid cells [16] to induce the classical Th2 cytokines IL-4, IL-5, and IL-13. In turn, IL-13 impairs barrier integrity and induces goblet cell hyperplasia and mucus production [3, 17].

IL-34 that has recently been described as an important interleukin in IBD is thought to promote the differentiation of M2 monocytes [18]. In normal wound healing processes, M2 macrophages are responsible for resolving inflammation and promoting fibrosis [19].

Thymic stromal lymphopoietin has been described as an important modulator of Th1/Th2 immune responses and to play a major role in tissue homeostasis and maintaining peripheral tolerance [20]. In the presence of IL-4, TSLP instructs dendritic cells to enforce a Th2 phenotype in naïve T-cells ultimately resulting in remodeling of the colon architecture [21–23]. In concert with IL-13 TSLP induces the differentiation of M2 monocytes [24] and fibrocytes [25] which evokes an immune modulatory and remodeling response by the secretion of TGFß1, IL-10, and periostin. TARC activates and attracts leukocytes bearing the CCR4 receptor to include Th2-, Th17-cells, and monocytes [26–28]. Recently it has been shown to signify effector-type FoxP3+ CD4+ regulatory T cells. Depletion of these cells with an anti CCR4 antibody evoked anti-tumor responses in humans [29].

Immune profiling of patients had previously identified CD1a expressing macrophages or monocytes as biomarkers to distinguish between UC patients and Non-UC donors [4]. CD1a has been known for decades as a phenotypic marker of human epidermal Langerhans cells. Like other members of the CD1 family, CD1a presents lipids to evoke T cell activation resulting in the release of IL-22, IL-13 and IFNy [8, 30]. IL-22 promotes wound healing by inducing epithelial hyperplasia [31]. CD1a macrophages activate conventional αß CD4+ T cells. Furthermore, CD1a expressing monocytes have been shown to be widely distributed in other organs including the colon and have been shown to express IL-12 and to activate Th1 cells [7, 32]. The supply of fatty acid ligands could be provided by phospholipase A2 (PLA2)—an major ingredient of inflammation and bee- and wasp venom [33].

The map of the disease network was validated by treating NSG-UC mice with therapeutics interfering with the ‘acute’ or ‘remodeling’ inflammatory condition. Results confirmed the previously suggested two inflammatory conditions and indicated that the disease map might be developed into a useful tool to predict efficacy of therapeutics and their potential mechanistic side effects.

## Methods

### Isolation of PBMC and engraftment

60 ml of peripheral blood were collected from the arm vein of patients suffering from UC in trisodium citrate solution (S-Monovette, Sarstedt, Nürnberg, Germany). Hank’s balanced salt solution (HBSS, Sigma Aldrich, Deisenhofen, Germany) was used to dilute the blood in a 1:2 ratio and 30 ml of the suspension were loaded onto LeucoSep tubes (Greiner Bio One, Frickenhausen, Germany). Peripheral blood mononuclear cells (PBMC) were separated by centrifugation at 400*g* for 30 min and no deceleration. The interphase was extracted and diluted with phosphate buffered saline (PBS) to a final volume of 40 ml. Cells were counted and centrifuged at 1400*g* for 5 min. The cell pellet was resuspended in PBS at a concentration of 4 × 10^6^ cells in 100 µl.

Six to eight-week old NOD.cg-Prkdc^SCID^ Il2rg^tm1Wjl^/Szj mice (abbreviated as NOD IL-2Rγ^null^) were engrafted with 100 µl cell suspension into the tail vein on day 1.

### Animal study protocol

NOD IL-2Rγ^null^ mice were obtained from Charles River Laboratories (Sulzfeld, Germany). Mice were kept under specific pathogen-free conditions in individually ventilated cages in a facility controlled according to the Federation of Laboratory Animal Science Association (FELASA) guidelines. Following engraftment (day 1) mice were pre-sensitized by rectal application of 150 µl of 10% ethanol on day 8 using a 1 mm cat catheter (Henry Schein, Hamburg, Germany). The catheter was lubricated with Xylocaine©Gel 2% (AstraZeneca, Wedel). The rectal application was performed under general anesthesia using 4% isoflurane. Post application mice were kept at an angle of 30° to avoid ethanol dripping. On day 15 and 18 mice were challenged by rectal application of 50% ethanol following the protocol of day 8. Mice were sacrificed on day 21. Pitrakinra (10 µg in 0.5% Methylcellulose, 0.05% TWEEN 80 in PBS) [34] was applied on day 7–9 and 14–21. Sterile Saline (B. Braun Melsungen AG, Germany) served as a control. Infliximab, [6 mg/kg (Remicade©, Janssen The Netherlands)] and isotype control (30 µg in 200 µl PBS, Morphosys AG, Planegg, Germany) were applied on day 7 and 14. All treatments were applied intraperitoneally.

### Clinical activity score

The assessment of colitis-severity was performed daily according to the following scoring system: Loss of body weight: 0% (0), 0–5% (1), 5–10% (2), 10–15% (3), 15–20% (4). Stool consistency: formed pellet (0), loose stool or unformed pellet (2), liquid stools (4). Behavior: normal (0), reduced activity (1), apathy (4) and ruffled fur (1). Body posture: intermediately hunched posture (1), permanently hunched posture (2). The scores were added daily into a total score with a maximum of 12 points per day. Animals who suffered from weight loss > 20%, rectal bleeding, rectal prolapse, self-isolation or a severity score > 7 were euthanized immediately and not taken into count. All scores were added for statistical analysis.

### Isolation of human leukocytes

To isolate human leukocytes from murine spleen, spleens were minced and cells filtrated through a 70 µl cell strainer (Greiner Bio-One, Frickenhausen) followed by centrifugation at 1400*g* for 5 min and resuspended in FACS buffer (1× PBS, 2 mM EDTA, 2% FCS). For further purification cell suspensions were filtrated using a 35 µm cell strainer (Greiner Bio-One, Frickenhausen) and then labeled for flow cytometry analysis. Cells were defined as shown in Additional file 1: Table S1.

### Flow cytometry analysis

Labeling of human leukocytes was performed according to Additional file 1: Table S2. All antibodies were purchased from Biolegend (San Diego, USA) and used according to the manufacturer’s instructions. Flow cytometry was performed using a BD FACS Canto II™ and analysed with FlowJo 10.1-Software (FlowJo LLC, Oregon, USA).

### Histological analysis

Samples from distal parts of the colon were fixed in 4% formaldehyde for 24 h, before storage in 70% ethanol and were routinely embedded in paraffin. Samples were cut into 3 µm sections and stained with haematoxylin and eosin (H&E). Epithelial erosions were scored as follows: no lesions (0), focal lesions (1), multifocal lesions (2), major damage with the involvement of basal membrane (3). Inflammation was scored as follows: infiltration of few inflammatory cells into the lamina propria (1), major infiltration of inflammatory cells into the Lamina propria (2), confluent infiltration of inflammatory cells into the Lamina propria (3), infiltration of inflammatory cells including tunica muscularis (4). Fibrosis was scored as follows: focal fibrosis (1), multifocal fibrosis and crypt atrophy (2). The presence of edema, hyperemia and crypt abscess was scored with one additional point in each case. The scores for each criterion were added into a total score ranging from 0 to 12. Images were taken with a Zeiss AxioVert 40 CFL camera. Figures show representative longitudinal sections in original magnification. In Adobe Photoshop CS6 a tonal correction was applied to enhance contrasts within the pictures.

### RNA analysis

#### RNA extraction and cDNA synthesis

Approximately 1 cm from distal parts of the colon was homogenized by using a TissueLyser LT (Qiagen, Hilden, Germany) and total RNA was extracted according to the manufacturer’s instruction using RNeasy Plus Universal Mini Kit (Qiagen, Hilden, Germany) and Chloroform (Sigma-Aldrich, St. Louis, MO, USA). No further treatment with DNase was needed since gDNA Eliminator Solution was included in the kit.

Five microgram of total RNA were used for cDNA synthesis. Reverse transcription was performed using a Mastercycler gradient (Eppendorf, Hamburg, Germany) using QuantiNova Reverse Transcription Kit (Qiagen, Hilden, Germany). Between 10 pg and 100 ng were used according to the TaqMan Fast Advanced Master Mix protocol (Thermo Fisher Scientific, Waltham, MA, USA).

RNA and cDNA purity determined by Nanodrop 2000 spectrophotometry (Thermo Fisher Scientific, Waltham, MA, USA).

#### Quantitative real-time PCR

Quantitative real-time PCR was performed according to the TaqMan Fast Advanced Master Mix protocol (Thermo Fisher Scientific, Waltham, MA, USA) according to the Applied Biosystems StepOnePlus real-time PCR system (Thermo Fisher Scientific, Waltham, MA, USA). included The following primers were used: The housekeeping genes GAPDH (Mm99999915_g1) and GUSB (Mm00446953_m1) as well as TGFβ (Mm01178820_m1), HGF (Hs04329698_m1), CCL17 (Mm01244826_g1), IFNγ (Hs00989291_m1) and TNFα (HS01113624_g1) (Single Tube TaqMan Gene Expression Assays, Thermo Fisher Scientific, Waltham, MA, USA). The analysis was performed using StepOnePlus™ Software v2.3.

The mean cycle threshold (CT) value was calculated for the housekeeping genes. Relative expression values for the analyzed genes were then calculated as the difference between the mean cycle threshold (CT) of the housekeeping genes and the analyzed gene (delta CT) and depicted as the logarithmic value.

#### RNAseq analysis

RNAseq and bioinformatic analyses were performed by IMGM Laboratories GmbH, Martinsried, Germany. RNA sequencing was performed on the Illumina TruSeq 500 next generations sequencing system and its high output mode with 1 × 75 bp single-end read chemistry. For all samples, the most recent mouse reference genome (mus musculus GRCm38.p3 C57BL/6, source: NCBI) as well as the most recent human reference genome hg19 (homo sapiens, GRCh 37.p5, source NCBI) were combined and used as reference sequence for mapping. Expression values were processed to total exon read count/mapped reads [million] * exon length [kb] (RPKM). The comparison of the control versus ethanol challenged group was based on the ‘total exon reads’ expression values and was analyzed statistically by the CLC Genomics Workbench tool ‘empirical analysis of DGE (EDGE)’. For comparison of the groups, the ‘Exact Test’ was used.

For analyzing differences in expression value of mouse genes a feature was classified as induced/repressed in a specific comparison if its FDR-corrected p value is ≤ 0.05 and if it has a fold change value ≥ 2/≤ 2.

For analyzing differences in expression value of human genes, a feature is classified as induced/repressed in a specific comparison if its non-corrected p value is ≤ 0.05.

### Statistics

Statistical analysis was performed with R: a language and environment for statistical computing (R Foundation for Statistical Computing, Vienna, Austria. URL https://www.R-project.org/) and BRB Array Tools (https://brb.nci.nih.gov/BRB-ArrayTools/). Variables were represented with mean, standard deviation, median, and IQR values. To compare binary groups A two-sided Student’s t test and a confidence level = 0.95 was used. If more than, two groups were analyzed ANOVA followed by Tukey HSD was conducted. Spearman correlation analysis was performed.

## Results

### Challenge with ethanol predominantly affects monocytes and macrophages in the NSG-UC mouse model

**Fig. 1:**
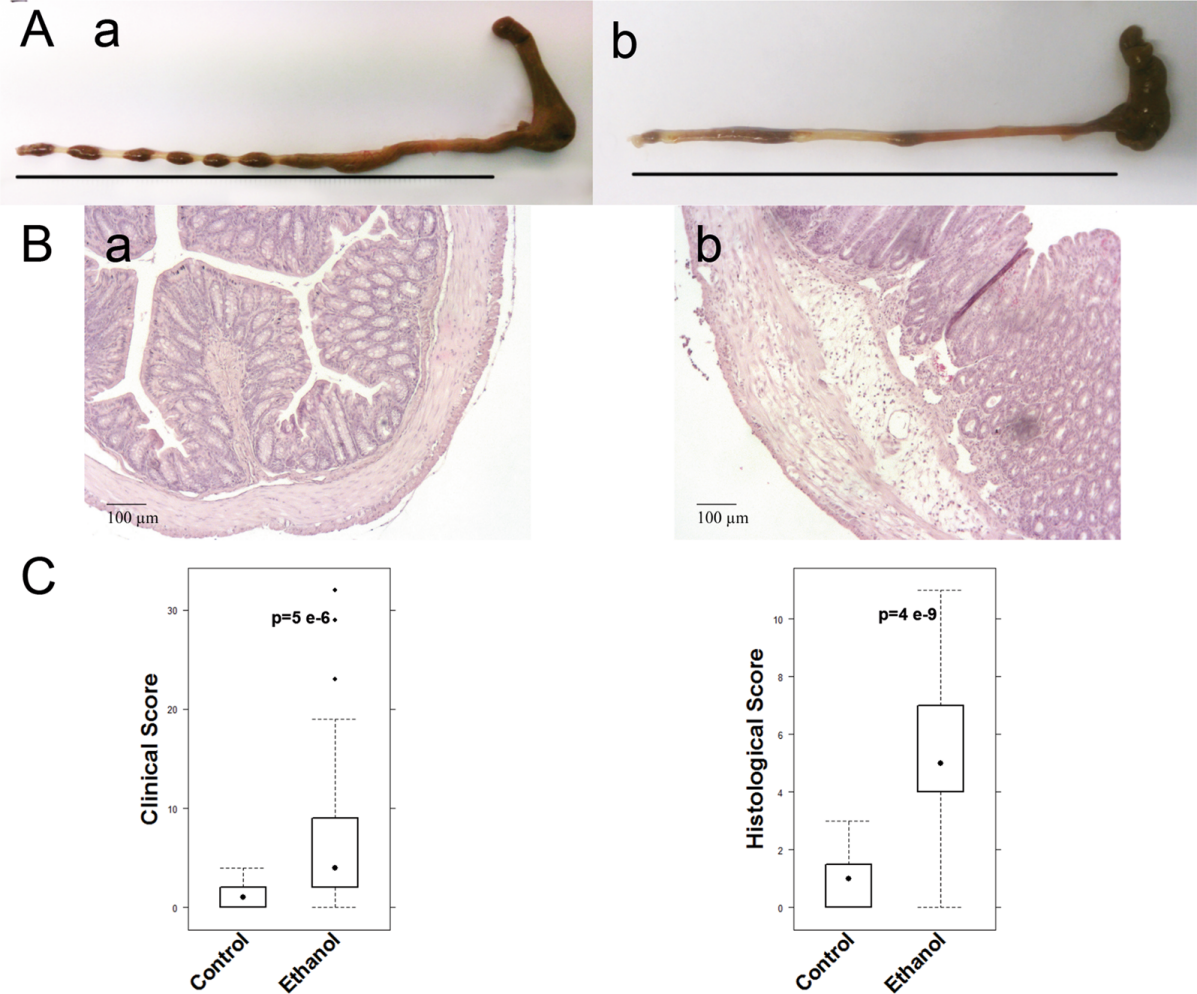
NSG mice reconstituted with PBMC from UC donors develop UC like symptoms and phenotype upon challenge with ethanol. **A** Macrophotographs of colons at autopsy of NSG-UC mice. **a** Unchallenged control group (control). **b** Group challenged with 10% ethanol at day 8, and 50% ethanol at days 15 and 18 (ethanol). **B** Photomicrographs of H&E-stained sections of distal parts of the colon from mice that had been challenged **a** control, **b** ethanol. Arrow indicates edema and influx of inflammatory cells. **C** Clinical- and histological score of control and ethanol challenged mice that had been challenged as described in **A** depicted as boxplot diagrams. Boxes represent upper and lower quartiles. Whiskers represent variability and outliers are plotted as individual points. Sample sizes: clinical activity score control n = 32, ethanol = 38; histological score: control n = 31, challenged n = 37. For comparison of unchallenged control group versus challenged group, a Student T-test was conducted

**Fig. 2:**
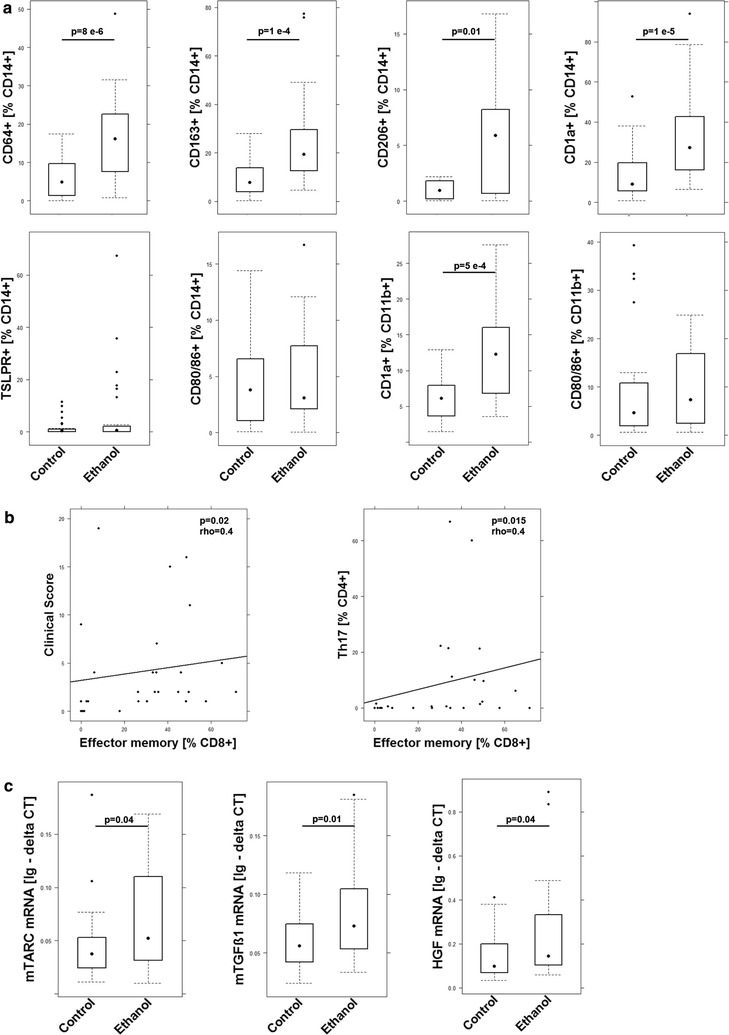
Inflammation induced by ethanol in NSG mice reconstituted with PBMC from UC patients is characterized by subtypes of monocytes, macrophages, effector memory CD8+ cells and by Mtarc, mTGFß1 and HGF. **a** Flow cytometric analysis of subtypes of CD14+ monocytes and CD11b+ macrophages of human leukocytes isolated from spleens. Unchallenged control group (control), group challenged with 10% ethanol at day 8 and 50% ethanol at days 15 and 18 (ethanol). For complete data set see Additional file 1: Table S3). Labels given on x-axes on the bottom row apply to all charts. **b** Correlation analysis of effector memory CD8+ T-cells with clinical activity score and Th17 T-cells depicted as scatter blots. Numbers indicate Spearman rank-order correlation coefficients (rho) and p values. Sample sizes: clinical score n = 29, Th17 n = 25. **c** mRNA expression analysis of mTARC, mTGFß1 and HGF depicted as boxplots. Lg-delta CT, logarithmic delta cycle threshold. Boxes represent upper and lower quartiles. Whiskers represent variability and outliers are plotted as individual points. For comparison of unchallenged control group versus challenged group, a Student T-test was conducted. Labels given on x- and y-axes on the bottom and the side row apply to all charts

To identify cellular and molecular markers that were correlated with inflammation in the NSG-UC mouse model experiments were repeated with eight different donors (Table 1). NSG mice were reconstituted with PBMC and challenged according to a standard protocol as described in “Methods”. Eight days post reconstitution the mice were divided into two groups: one was left unchallenged, the other was challenged by rectal application of ethanol. The groups contained four to six animals. Upon challenge with ethanol mice stools became soft or liquid, the animals lost weight, and the activity was reduced. Control animals displayed hardly any symptoms. Symptoms were classified according to a clinical score described in “Methods”. Upon challenge, the clinical score increased significantly (Fig. 1C), (for complete data set see Additional file 1: Table S3). Macroscopic inspection of colons corroborated the data obtained from analyzing the clinical score. The ethanol challenged mice were characterized by soft or absent pellets and dilated colons (Fig. 1A). Histological analysis revealed the influx of a mixed infiltrate of leukocytes, edema, crypt loss, and changes in the colon architecture (Fig. 1B). To further analyze the inflammation human leukocytes were isolated from the spleen and subjected to flow cytometric analysis. Although we would identify donor dependent patterns on subjects of T-cells and B-cells (data not shown) the most donor independent effects were observed in macrophages and monocytes and subtypes thereof (Fig. 2a). Thus, we would define CD14+ monocytes expressing CD64, CD163, CD206 or CD1a and CD11b+ macrophages expressing CD1a as the most significant cell types associated with inflammation in the NSG-UC model. We did not observe increased maturation of macrophages and monocytes as indicated by similar levels of CD86 expressing macrophages and monocytes. Effects on subsets of T-cells were donor dependent and could not be described as generally affected by challenge, however, we could observe a correlation of effector memory CD8+ cells with the clinical activity score (rho = 0.4, p = 0.02) and with Th17 CD4+ T-cells (rho = 0.4, p = 0.015), indicating that both cell types might be involved in the development of symptoms and phenotype (Fig. 2b). mRNA expression analysis by RT PCR from RNA isolated from distal parts of the colon revealed that TARC, TGFß1, and HGF were the markers most reliably associated with inflammation. IFNγ levels could not be detected in all samples and displayed high variability (Fig. 2c).

**Table 1:** Patient baseline characteristics

Donor	Treatment	SCCAI	Age	Gender	Time since diagnosis
1	Infliximab, glucocorticoid, mesalazine	5	36	w	12
2	No	10	52	m	21
3	No	5	52	m	21
4	Colectomy, loperamid, mesalazine,		46	w	20
5	Infliximab, glucocorticoid, mesalazine	11	36	w	12
6	Adalimumab, mesalazine	5	35	m	16
7	Infliximab	2	68	w	38
8	Adalimumab	3	53	w	13

### RNA expression analysis

To gain a better understanding of the epithelial triggers eliciting the immunological responses in the NSG-UC mouse model in response to challenge with ethanol, an RNAseq analysis was performed with tissue samples isolated from distal parts of the colon. For reconstitution, a donor was selected who exhibited a simple clinical activity score index (SCCAI) [35] of 5. Reconstitution and challenge was performed according to the standard protocol. Following reconstitution, mice were separated into two groups: the control group (control) and the ethanol challenged group (ethanol). Each group contained four animals. Upon challenge with ethanol the ethanol group developed a clinical activity score of 5 ± 2.9, (mean ± S.D.) and a histological score of 6.25 ± 2.2, whereas the control group developed hardly any symptoms and phenotype as indicated by a clinical activity score of 1.5 ± 1.3 and a histological score of 1.5 ± 0.6. Table 2 shows a selection of prominent proteins whose expression was significantly changed upon challenge with ethanol (complete analysis will be made accessible online). Unexpectedly, expression of IL-33 was decreased by a factor of almost 2, whereas expression of IL-34 was significantly increased. Expression of IL-17c was also increased by a factor of 11, although the original values were much lower as compared to IL-33 and IL-34. Expression of TSLP was increased by a factor 1.39. As previously shown TGFß1 was elevated by a factor of 1.45 in response to ethanol and in this experiment TNFa seemed to be a major proinflammatory cytokine. No expression of hIFNγ could be detected in this experiment a result which was also confirmed by RT-PCR analysis (data not shown). Unexpectedly, an antagonist of IL-1b, IL-1rn was significantly expressed and elevated in response to ethanol (fold change = 1.39). The inflammation induced by ethanol was also characterized by the typical inflammatory markers of variants of phospholipases A2 Pla2g10 and Pla2g2f which were elevated 1.16 and 1.33 fold, respectively and metalloproteinases Mmp14 and 2 which were elevated by a factor of 1.53 and 1.3, respectively. Challenge with ethanol also affected cell survival and apoptosis as indicated by elevated expression of Casp7 (1.16 fold change), Card9 (1.64 fold change), S100a14 (1.29 fold change) and S11a16 (1.21 fold change). Furthermore, challenge with ethanol induced the expression of Pparg, indicating a strong metabolic effect. In this experiment, the growth factors affected by ethanol were fibroblast growth factor (FGF) 11 and 9 and insulin growth factor (IGF) 1, whereas HGF was expressed, but expression was not induced (data not shown). The observed fibrosis was confirmed by induced expression of collagen (col) 1a1, which was elevated 2.26 fold. In summary, expression analysis suggests that IL-34 is the main cytokine released by epithelial cells to induce proliferation and differentiation of monocytes and macrophages. This result is in agreement with the previous observations showing that the predominant effect of ethanol challenge was observed with monocytes and macrophages.

**Table 2:** Challenge with ethanol resulted in increased expression gene associated with inflammation

Feature ID	Range	IQR	Difference	Fold change	p value
Cytokine					
Il17c	7	2	2.5	11	0.011
Il1rn	3090	1724	718.75	1.29	0.002
Il33	712	483	− 347	− 1.98	0.002
Il34	326	132	81.5	1.44	0.007
Il7	179	120	39.5	1.20	0.027
Tgfb1	243	70	63.25	1.45	0.036
Tnf	100	57	49.25	4.65	2E−07
Tslp	39	22	10.5	1.39	0.062
Growth factor					
Fgf11	248	71	− 95.75	− 1.65	0.013
Fgf9	111	83	− 57	− 1.57	0.018
Igf1	1152	431	412	1.91	1E−04
Phospholipase					
Pla2g10	2170	1285	341.25	1.16	0.039
Pla2g2f	1314	496	349.75	1.33	0.005
ECM					
Col1a1	11,375	2581	4058	2.26	1E−08
Metalloproteinase					
Mmp14	2092	312	533.5	1.53	0.010
Mmp2	3247	1104	611.5	1.30	0.045
Cell survival/apoptosis					
Casp7	2691	1749	468	1.16	0.028
Card9	35	10	9	1.64	0.044
S100a14	4618	1785	1125.5	1.29	0.002
S100a16	5359	1594	924	1.21	0.033
Metabolism					
Pparg	1021	374	354	1.54	7E−05

Sections from distal parts of the colon of NSD mice reconstituted with PBMC derived from a patient with UC were challenged with ethanol and subjected to RNAseq analysis. Unchallenged control (control, n = 4), group challenged with 10% ethanol at day 8, and 50% ethanol at days 15 and 18 (ethanol, n = 4). Expression values are depicted as mean total exon read count/mapped reads [million] * exon length [kb] (RPKM). The difference between the highest and the lowest expression value over all samples (range), inter-quantile rage of expression values (IQR), difference between the mean of expression values of the ethanol challenged group and the control group, mean expression value of ethanol challenged group divided by the mean expression value of the control group, FDR corrected p value (EDGE test) (p value)

### Design of disease network

**Fig. 3:**
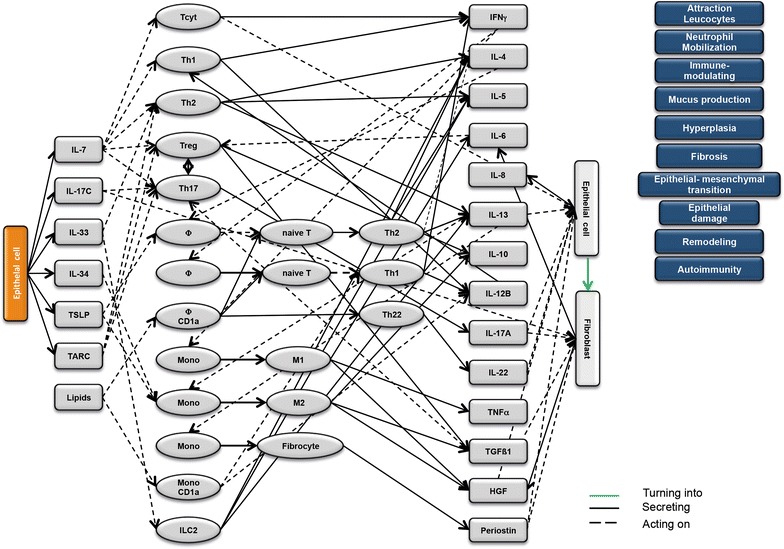
Design of disease network based on mRNA expression analysis and effects of cytokines, chemokines and growth factors described in the literature. No signal from epithelial cells

**Fig. 4:**
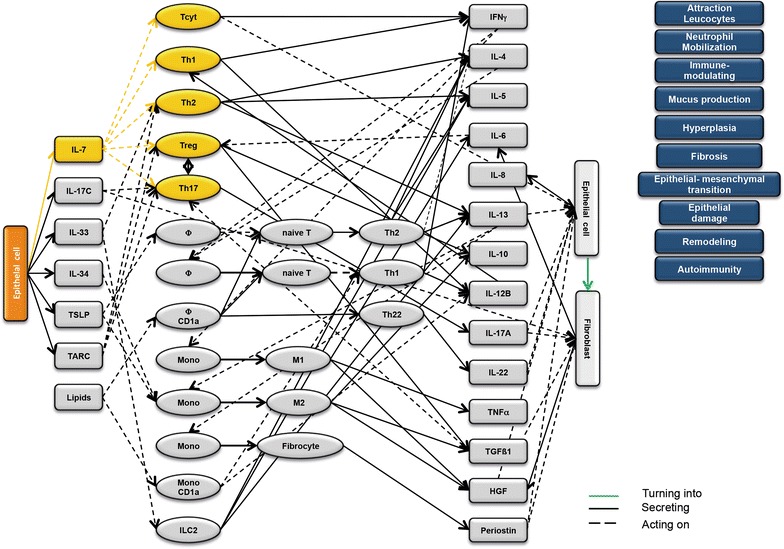
Design of disease network based on mRNA expression analysis and effects of cytokines, chemokines and growth factors described in the literature. Activation of proinflammatory pathway by IL-7

**Fig. 5:**
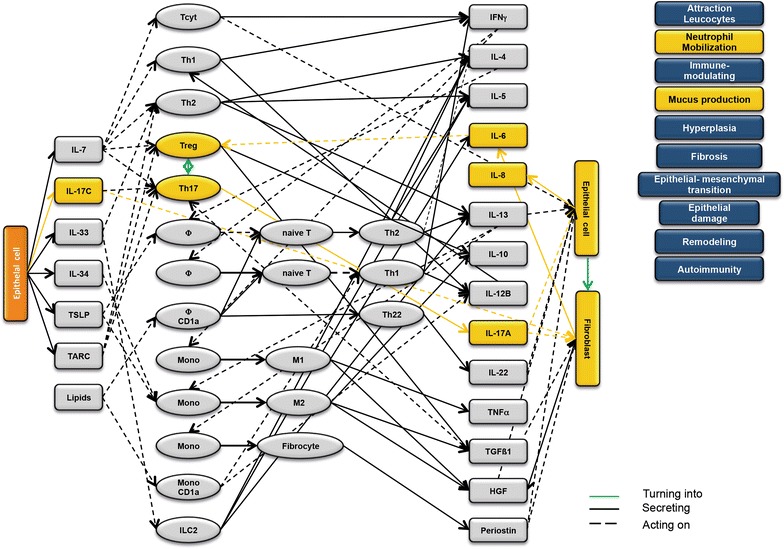
Design of disease network based on mRNA expression analysis and effects of cytokines, chemokines and growth factors described in the literature. Activation of proinflammatory pathway by IL-17c

**Fig. 6:**
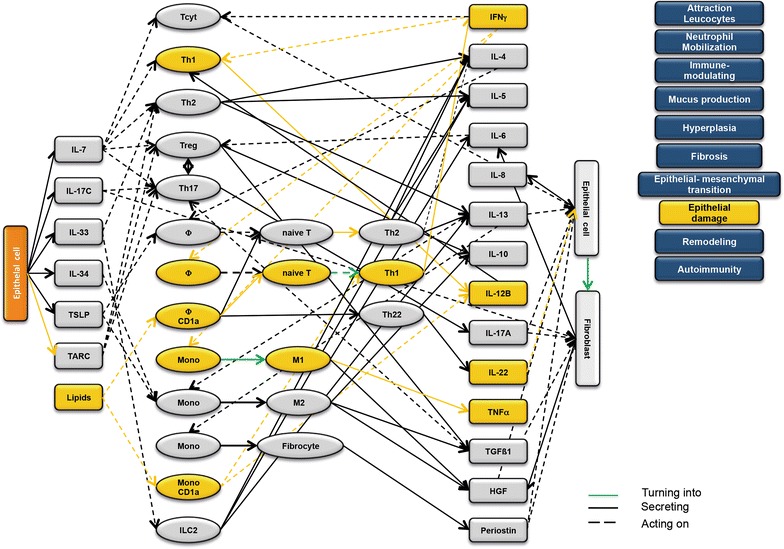
Design of disease network based on mRNA expression analysis and effects of cytokines, chemokines and growth factors described in the literature. Potential pathway induction by lipids

**Fig. 7:**
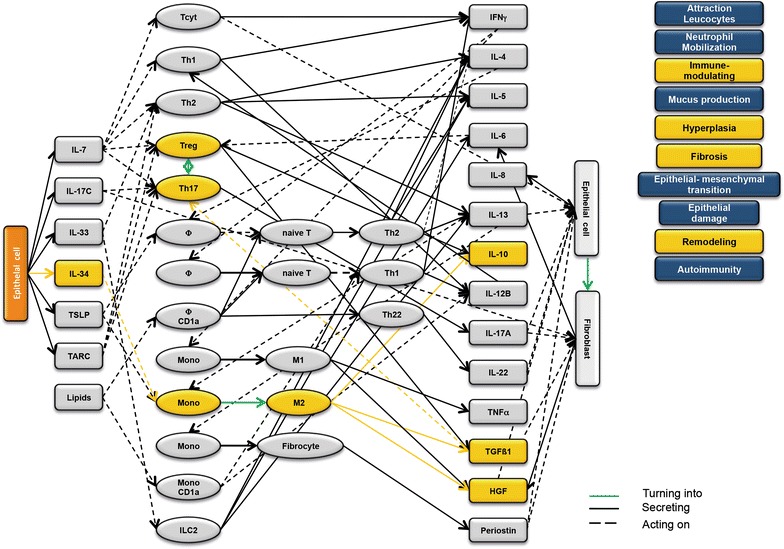
Design of disease network based on mRNA expression analysis and effects of cytokines, chemokines and growth factors described in the literature. Activation of remodeling pathway by IL-34

**Fig. 8:**
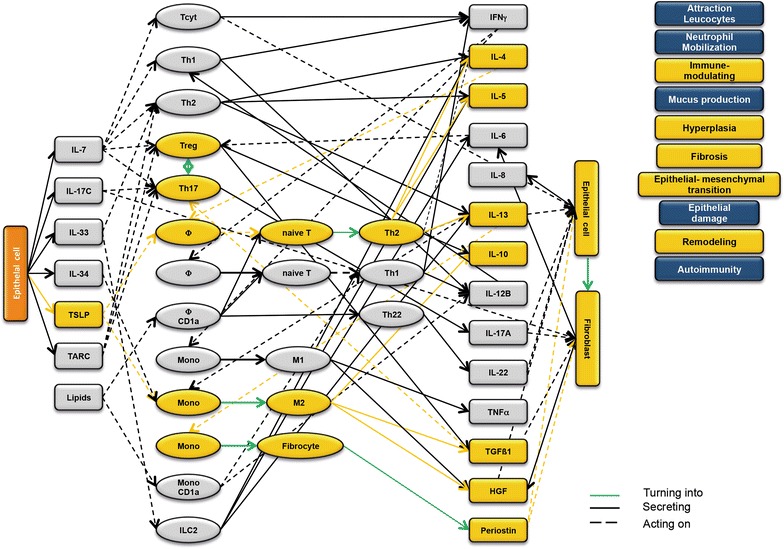
Design of disease network based on mRNA expression analysis and effects of cytokines, chemokines and growth factors described in the literature. Stimulation of remodeling pathway by TSLP

**Fig. 9:**
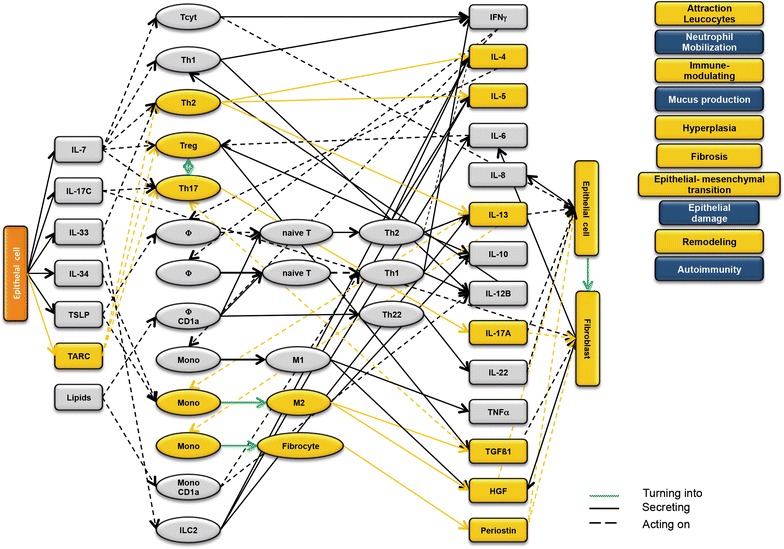
Design of disease network based on mRNA expression analysis and effects of cytokines, chemokines and growth factors described in the literature. Attraction and stimulation of remodeling pathway by TARC

**Fig. 10:**
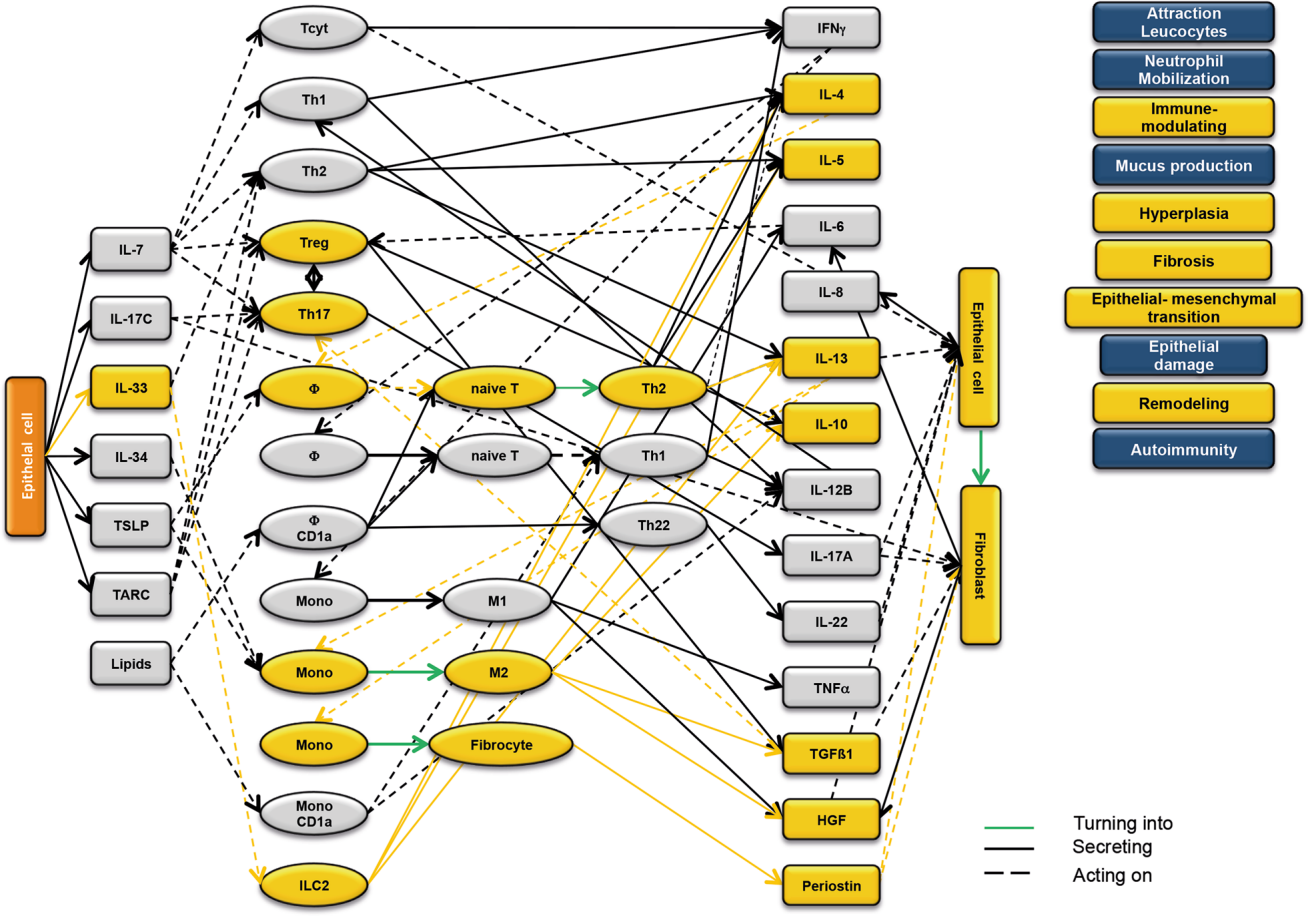
Design of disease network based on mRNA expression analysis and effects of cytokines, chemokines and growth factors described in the literature. Activation of remodeling pathway by IL-33

Based on the results obtained from the RNAseq analysis and the effects of interleukins, chemokines and growth factors described in the literature, a disease network was designed that might lead to a better understanding of inflammatory processes in the NSG-UC model (Fig. 3). Whereas IL-7 is a general activator of T cells (Fig. 4), IL-17c might be responsible for a proinflammatory response resulting in increased levels of IL-8, IL-6 and IL-17A ultimately leading to increased mucus production and attraction of neutrophils (Fig. 5). Lipids released by PLA2 and presented by CD1a expressing monocytes and macrophages might induce autoimmunity by activation Th1 (our own results) or Th22 cells (Fig. 6) [6–8]. The inflammatory remodeling condition would be the result of the Th2 driven inflammation induced by IL-34, TSLP and TARC (Figs. 7, 8, 9). In UC patients, this inflammatory condition might be responsible for fibrosis and epithelial hyperplasia. It is noteworthy that levels of IL-33 which has been shown to play a crucial role in atopic dermatitis [16] are decreased indicating that ILC2 might not be drivers of the remodeling condition in the NSG-UC (Fig. 10).

As previously suggested, this disease network shows two arms of inflammation evoked upon damage of the mucosa: A Th1/M1 Tcyt characterized inflammation, which we would refer to as acute inflammatory, and a Th2/M2 Treg characterized inflammation, which we previously referred to as the remodeling condition. A patient in relapse would most probably exhibit both arms of inflammation.

### Validation of the disease network in the NSG mouse model

**Fig. 11:**
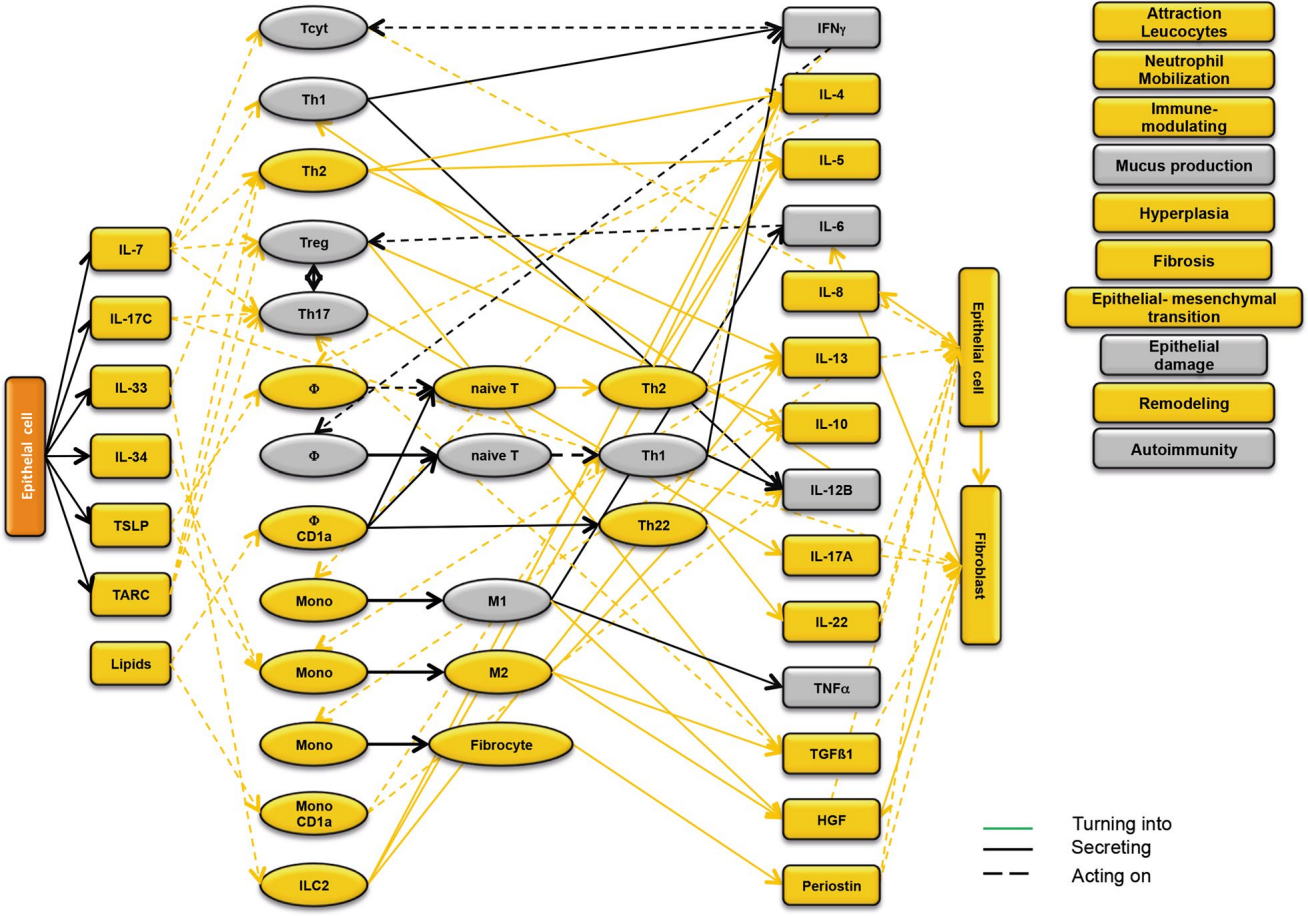
Design of disease network based on mRNA expression analysis and effects of cytokines, chemokines and growth factors described in the literature. Blockade of TNFα leads to inhibition of the proinflammatory pathway whereas the remodeling pathway remains unaffected

**Fig. 12:**
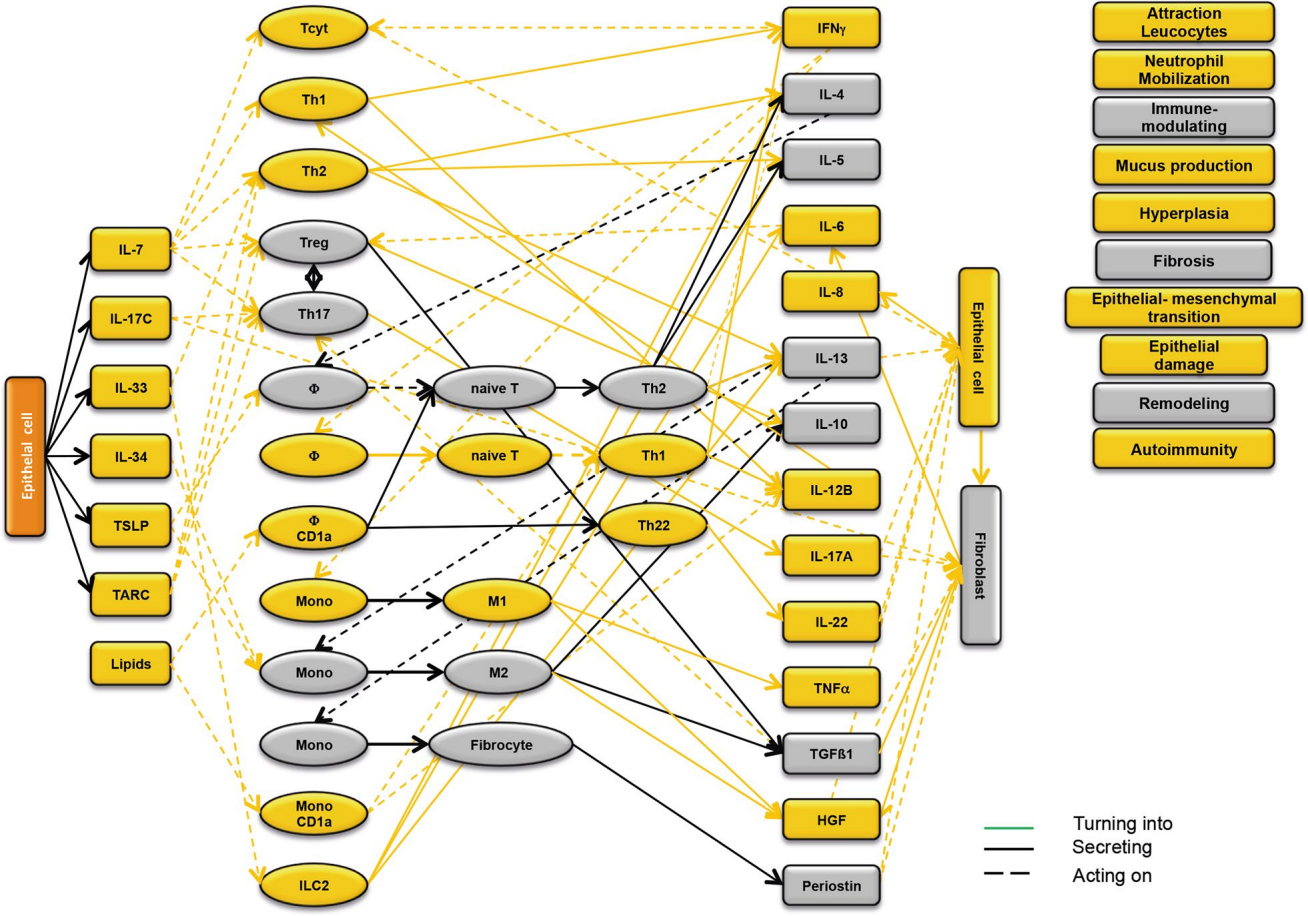
Design of disease network based on mRNA expression analysis and effects of cytokines, chemokines and growth factors described in the literature. Blockade of IL-4Rα1 receptor leads to inhibition of the remodeling pathway and promotes the proinflammatory pathway

**Fig. 13:**
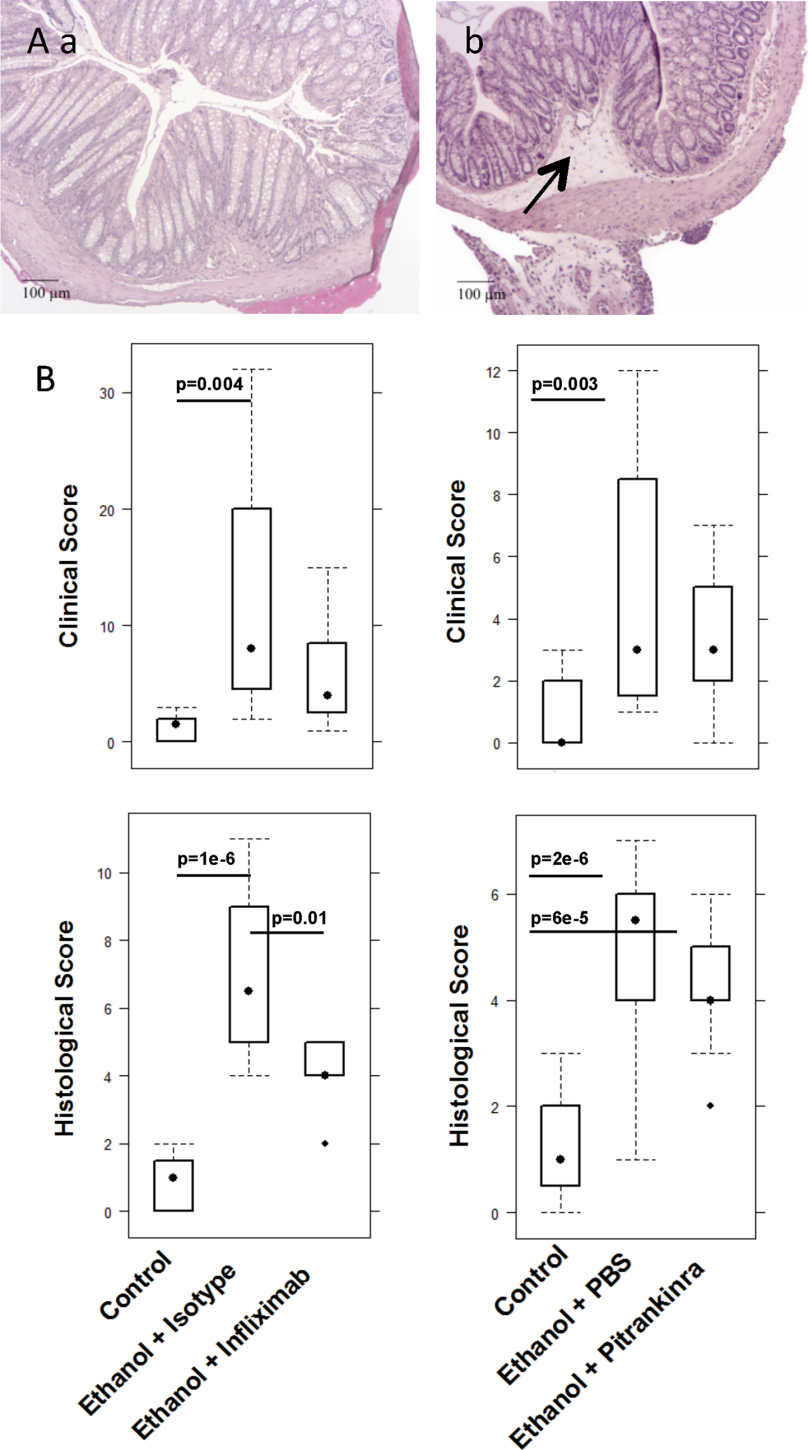
Treatment infliximab or pitrakinra had different effects in NSG mice reconstituted with PBMC from UC patients. **A** Photomicrographs of H&E-stained sections of distal parts of the colon from mice that had been reconstituted with PBMC from patients with UC, challenged with ethanol and treated with **a** infliximab, **b** pitrakinra. Mice were treated by intraperitoneal application of pitrkinra on days 7–9 and 14–21 and PBS were used as carrier control. Infliximab was applied by intraperitoneal injection on day 7, 14, 17 and isotype was used as a control. Arrows indicate edema and influx of inflammatory cells. **B** Depiction of clinical- and histological score as boxplots. Boxes represent upper and lower quartiles, whiskers represent variability and outliers are plotted as individual points. Unchallenged control (control), challenged control (ethanol + PBS/isotype), challenged and treated (ethanol + infliximab/pitrakinra). For comparison of groups ANOVA followed by TukeyHSD was conducted. Labels given on y-axes on the bottom apply to all charts

**Fig. 14:**
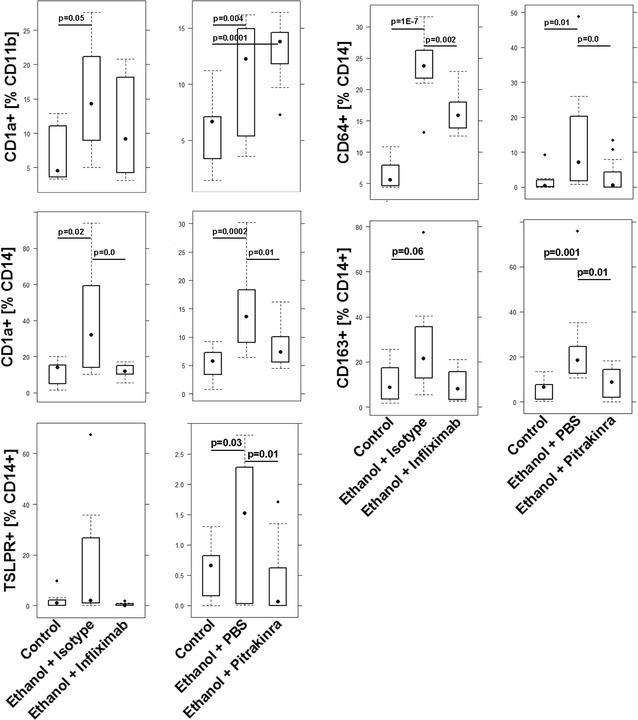
Treatment infliximab or pitrakinra had different effects in NSG mice reconstituted with PBMC from UC patients. Flow cytometric analysis of frequencies of subtypes of CD11b+ macrophages and CD14+ monocytes depicted as boxplots. Mice were treated as described in Fig. 13. For complete data set see Additional file 1: Tables S4, S5). Boxes represent upper and lower quartiles, whiskers represent variability and outliers are plotted as individual points. Unchallenged control (control), challenged control (ethanol + PBS/isotype), challenged and treated (ethanol + infliximab/pitrakinra). For comparison of groups ANOVA followed by TukeyHSD was conducted. Labels given on x- and y-axes on the bottom and the side row apply to all charts

**Fig. 15:**
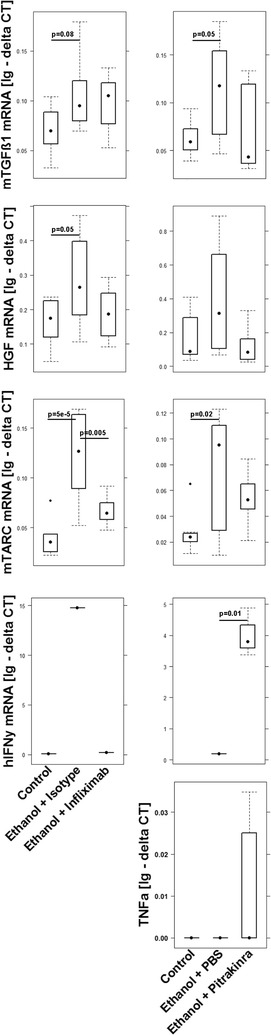
Treatment infliximab or pitrakinra had different effects in NSG mice reconstituted with PBMC from UC patients. mRNA expression levels of TGFß1, HGF, TARC, IFNγ and TNFa as boxplots. Mice were treated as described in Fig. 13. For complete data set see Additional file 1: Tables S4, S5). Lg-delta CT, logarithmic delta cycle threshold. Boxes represent upper and lower quartiles, whiskers represent variability and outliers are plotted as individual points. Unchallenged control (control), challenged control (ethanol + PBS/isotype), challenged and treated (ethanol + infliximab/pitrakinra). For comparison of groups ANOVA followed by TukeyHSD was conducted. Labels given on x- and y-axes on the bottom and the side row apply to all charts

To examine whether the NSG-UC model can be used to validate the disease map, two therapeutics were tested, which were expected to target crucial immunological junctions. PBMC were isolated from patients in relapse. NSG mice were reconstituted and challenged according to a standard protocol described in “Methods”. Seven days post reconstitution the mice were divided into three groups: one was left unchallenged, one was challenged by rectal application of ethanol plus the respective carrier, one was challenged and additionally treated with the respective therapeutic. Each group contained four animals. Experiments were repeated with two different donors in relapse (SCCAI > 5) (for patients baseline demographics see Table 1). The response to treatment was compared to the control group and the ethanol challenged group (for complete data set, please see Additional file 1: Tables S4, S5). As observed in previous experiments control animals remained unaffected, whereas in all ethanol-challenged groups mice developed the previously described symptoms and phenotype [11]. At first, infliximab was tested, which is supposed to suppress the acute arm of inflammation by depletion of TNFα bearing M1 monocytes. However, as suggested by the analysis of inflammatory profiles of human UC patients and as predicted by the disease network (Fig. 11) one would expect that in mice the remodeling inflammation would be retained. As shown in Fig. 13B mice benefited from the treatment indicated by the clinical- and histological score. Analysis of the histological sections revealed, however, fibrotic alterations of the colon architecture (Fig. 13Aa). The previously identified acute inflammatory markers CD11b+ CD1a+, CD14+ CD1a+, CD14+ CD64+, TARC, HGF and IFNγ diminished, whereas expression of TGFß1 was not affected, corroborating the histological results (Figs. 14, 15). Infliximab treatment also affected M2 monocytes as indicated by a decrease of CD14+ CD163+ monocytes, suggesting that the fibrotic changes not solely rely on M2 monocytes. These results corroborated results from a previous study which has shown that treatment of patients with infliximab induces inflammatory remodeling condition [4]. Next, we tested a therapeutic targeting monocytes bearing the IL-4 Rα receptor, which is supposed to be predominantly expressed on M2 monocytes and Th2 cells. Pitrakinra, which has been described as an inhibitor of IL-4Rα/IL-2Rγ and IL-4Rα/IL-13Rα1 receptor complexes is thus supposed to inhibit Th2/M2 directed inflammation without suppressing the Th1/M1. According to the disease network, one would expect that NSG-UC mice would not benefit from the treatment with pitrakinra (Fig. 12). Histological analysis of H&E stained revealed severe damage to the colon architecture and influx of inflammatory cells (Fig. 13Ab). As shown in Fig. 13B, the clinical- and the histological score increased as well as the inflammatory markers CD11b+ CD1a+ and IFNγ (Figs. 14, 15) (for complete data set see Additional file 1). Mice also exhibited increased expression of TNFα, suggesting that the inflammatory balance is shifted towards a Th1 response. The fact that levels of TGFß1 and frequencies of CD14+ CD163+ monocytes were decreased supported the hypothesis that the remodeling arm of the inflammation was suppressed in favor of the acute inflammation. However, frequencies of CD14+ CD1a+, CD14+ CD64+, CD14+ TSLPR+ monocytes were also affected, suggesting that IL-4Rα receptor is more widely expressed further corroborating the plasticity of monocytes but also suggested that suppressing IL4-Rα1 bearing monocytes is not sufficient to suppress symptoms and phenotype.

## Discussion

By now, the NSG-UC model has been proven as a robust animal model partially reflective of the human disease. Although it has not yet been shown how the toxic effects of ethanol translate into inflammation, we hypothesized that ethanol damages the mucosal epithelial lining and thereby induces the release of cytokines, chemokines and growth factors to counteract the potential harm accompanying the breach of the epithelial lining. The ultimate goal of the inflammatory process would thus be to defend the organisms against invading pathogens and the restoration of barrier integrity. Hence, the inflammation in the NSG-UC model would partially resemble a wound healing process. Wound healing processes are characterized by an influx of neutrophils, macrophages, and monocytes. According to a new concept, macrophages are considered accessory cell types which support their client cells—notabene mucosal epithelial cells in the gut- with various functions [36]. Clearance of apoptotic cells is one of the major functions of, however, macrophages have also the capacity to adapt to the inflammatory milieu and to direct to direct inflammation towards inflammatory, healing or regulatory. If our hypothesis is correct, one would assume a central role of macrophages and monocytes in the NSG-UC model. Although frequencies subtypes of T-cells, B-cells and NK T cells were found to be elevated in some experiments, these responses seemed patient dependent and could not be observed in all studies. However, the challenge with ethanol was found to profoundly affect monocytes and macrophages. Hence, we would define M1-, M2-, and CD1a expressing CD14+ monocytes and CD1a expressing CD11b+ macrophages as cell types associated with the ethanol-induced inflammation. Frequencies of all these cells types were highly correlated with the clinical activity-and histological score, however, this observation does not necessarily mean that these cell types cause or add to the pathology and phenotype of colitis like symptoms as M2 monocytes, e.g. are instrumental in controlling inflammation. Challenge with ethanol did not induce further maturation of macrophages and monocytes as indicated by even levels of CD11b+ CD86+ macrophages and CD14+ CD86+ monocytes in the unchallenged versus the challenged groups. In addition to macrophages and monocytes, a positive correlation was detected between effector memory CD8+ cells and the clinical score, corroborating previous results where CD44+ experienced T-cells were found to be elevated in the spleen as well as the colon of NSG-UC mice. This observation suggests that the onset of epithelial damage and inflammation might also be caused by cytotoxic T-cells. Furthermore, frequencies of effector memory CD8+ cells correlated positively with Th17 T-cells, suggesting that these cells also play a role in this inflammation. These results are in agreement with data obtained from UC patients where aberrant expression of circulating Th17, Th1 and cytotoxic T-cells was found in patients with UC [5]. The most reliable factors associated with the inflammation were the chemokine TARC, the cytokine TGFß1 and the growth factor HGF, all of which are considered hallmarks of wound healing processes [37]. The extent of inflammation was donor-dependent. Patients visiting our clinic are in most cases severely diseased and experience a chronic continuous form of the disease. A previous study has shown (our data) that the majority of these patients exhibit high level-expression of autoantibodies directed against immune cells indicating that loss or breach of tolerance may be one driver in UC. This observation was further corroborated by the detection of elevated levels of antigen experienced CD44 expressing CD8+ cells colons of UC patients as compared to Non-UC donors [4]. This observation might explain why the NSG-UC model requires PBMC from UC patients [11] and the positive correlation of CD8+ effector memory T-cells with the clinical score. NSG mice reconstituted with PBMC from healthy subjects did not experience colitis like symptoms and phenotype in response to ethanol.

As the previously analyzed factors expressed in the colon of NSG mice were selected by the wound healing hypothesis and hence the selection had a bias, an RNAseq analysis was performed. This analysis identified IL-7, IL-17c, IL-34 and TSLP as elevated upon challenge with ethanol all of which are expressed by epithelial cells. Whereas IL-7 is a general activator of T-cells, IL-17c is considered to promote inflammation [12–14]. In contrast, IL-34 and TSLP are thought to favor a remodeling of the colon architecture [18, 23, 24]. Also, phospholipase A2 was found to be elevated, suggesting that lipids presented by CD1a might also provoke inflammation. Rather unexpectedly, IL-33 levels were found to be decreased, indicating that a prominent route leading to remodeling is lacking in this model. Based on these results, the RT-PCR analysis from distal parts of the colon and mode of action of various cell types, chemokines, cytokines and factors described in the literature a disease network was designed. Highlighting various routes of inflammation induced by different cytokines, chemokines and lipids corroborated the previous findings that at least two inflammatory conditions prevail: a proinflammatory or acute condition and a remodeling condition. In a healthy organism, the acute inflammatory condition would ensure the protection against invading pathogens. In UC patients, the cause of the proinflammatory condition still remains to be elucidated. One possible explanation is that a breach of the epithelial lining leads to influx of bacteria into the mucosa. Alternatively, a general loss of tolerance towards the microbiota and the self-antigens might shift the intestinal immunological equilibrium. Our results suggest that there could be at least two drivers of the acute inflammatory process: IL-17c and CD1a expressing monocytes. In wound healing processes this equilibrium would be regained by restoring the integrity of the epithelial barrier and by mechanism to resolve inflammation. Our observations suggest that fibrosis in UC patients is the result of an ongoing restoration process to heal the destruction induced by the proinflammatory response. To understand the disturbed inflammatory balance future research has to examine why some UC patients have the capacity to control inflammation and experience times of remission while others do not.

Both types of inflammation would be expected to be induced upon challenge with ethanol in the NSG-UC mouse model. To validate this disease network, two different therapeutic to affect M1 or M2 macrophages were tested in the NSG-UC model. In a previous study, infliximab had been shown to induce a remodeling condition in UC patients [4]. The responses to infliximab in the NSG-UC model partially reflected this condition. Mice responded to treatment with decreased clinical activity and decreased frequencies of CD14+ monocytes, suggesting that not only M1 monocytes bear surface TNFα. Factors associated with inflammation such as TARC-, HGF- and INFγ mRNA decreased, whereas TGFß1 mRNA levels were not affected, suggesting an ongoing remodeling of the colon. This observation was corroborated by histological analysis that displayed less influx of inflammatory cells but retained fibrosis. Pitrakinra is also supposed to act on monocytes; in this case, however, monocytes bearing the IL-4Rα/IL-13Rα1 receptor complex would be targeted. Also, pitrakinra has been shown to suppress the differentiation of Th2 cells which carry the IL4-Rα/IL2Rγ chain. As observed when mice were treated with infliximab all subtypes of monocytes were affected, in this case, however, an increase of the CD1a expressing CD11b+ macrophage was observed. The increased clinical- and histological score increased levels of IFNγ and TNFα suggested that inflammation was completely shifted towards a Th1 phenotype, suppressing the remodeling arm of the inflammation.

Obviously, the disease network presented in this study is still too simple to completely describe the inflammatory dynamic underlying the pathology of UC. It does not yet include important aspects promoting inflammation as the role of B-cells, mast cells, basophils and eosinophils and the high plasticity of T-cells. It also does not yet account for concentration levels of cytokines, chemokines and growth factors and their cognate receptors. Hence, it is a first approach to portray inflammation in UC as a mobilée, in which the entire balance can be shifted when single cell types are depleted or promoted. It also suggests that it is not as simple to frame a certain cell type or cytokine to cause pathology based on the mere fact that frequencies or levels are elevated. Our model might explain why anrukinzumab, an anti-IL-13 monoclonal antibody was found to not affect clinical activity score, mucosal healing, rectal bleeding or clinical remission rates [38]. Conversely, it might explain why infliximab is contraindicated in patients with stenosis. Thus, every therapeutic can shift the balance and might have to be complemented with another therapeutic to reach full response. In the future, more comprehensive studies have to be performed to improve our knowledge of disease networks and the dynamics underlying inflammation in UC.

Obviously, the inadequacy to fully reflect the human disease also applies to the NSG-UC model. So far, it disregards the influence of basophils, mast cells, and eosinophils. Also, one has to keep in mind that it is a chimeric model and that some of the chemokines and cytokines do not exert their activity on receptors of the other species as it has been shown for IL-4 and TSLP [39, 40]. Thus, this model has to be improved in future studies to analyze the involvement of T-cells, B-cells basophils, eosinophils and mast cells. However, it seems to reflect the autoimmune aspect of the disease and can be used to validate therapeutics and hypothesis.

## Conclusions

The combination of patient profiling, the design of a disease network helps and the NSG-UC animal model may help to get a better understanding of the inflammatory processes in UC and may ultimately lead to individualized and phase dependent therapies.

## Additional file

**Additional file 1: Figure S1.** Gating strategy for human leukocytes isolated from mouse spleen. **Table S1.** Cellular markers used to define immune cells. **Table S2.** Monoclonal antibodies used in labelling of surface markers of leukocytes. **Table S3.** Data set of variables changed upon challenge with ethanol in the NSG-UC mouse model. **Table S4.** Data set of variables changed upon challenge with ethanol and treated with infliximab. **Table S5.** Data set of variables changed upon challenge with ethanol and treated with pitrakinra.

## Abbreviations

Card: caspase recruitment domain-containing protein
Casp: caspase
Col: collagen
CCR: chemokine receptor
FELASA: Federation of Laboratory Animal Science Association
FGF: fibroblast growth factor
HGF: hepatic growth factor
IFN: interferon
IGF: insulin growth factor
IL: interleukin
Mmp: matrix metallo protease
NSG: NOD-SCID IL2rγ^null^
NSG-UC: NOD-SCID IL2rγ^null^ reconstituted with PBMC from UC patients
PBMC: peripheral blood mononuclear cells
PBS: phosphate buffered saline
PLA2: phospholipase A2
Pparg: peroxisome proliferator-activated receptor gamma
RPKM: total exon read count
SCCAI: simple clinical activity score index
TARC: thymus and activation regulated chemokine
TGFß: tumor growth factor
Th: T helper cell
TNF: tumor necrosis factor
Treg: regulatory T cell
TSLP: thymic stromal lymphopoietin
TSLPR: thymic stromal lymphopoietin receptor
