# Supplementary material for: Design and validation of a disease network of inflammatory processes in the NSG-UC mouse model
Source: J Transl Med. 2017 Dec 28;15:265. doi: 10.1186/s12967-017-1368-4 (PMC5745765; doi:10.1186/s12967-017-1368-4)
Supplement: Supplementary file 1 — Additional file 1: Figure S1. Gating strategy for human leukocytes isolated from mouse spleen. Table S1. Cellular markers used to define immune cells. Table S2. Monoclonal antibodies used in labelling of surface markers of leukocytes. Table S3. Data set of variables changed upon challenge with ethanol in the NSG-UC mouse model. Table S4. Data set of variables changed upon challenge with ethanol and treated with infliximab. Table S5. Data set of variables changed upon challenge with ethanol and treated with pitrakinra. [file 12967_2017_1368_MOESM1_ESM.docx]

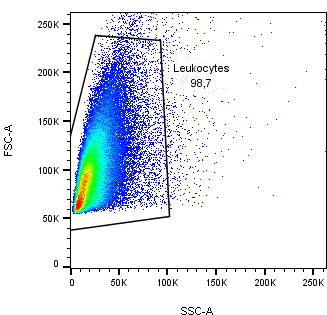

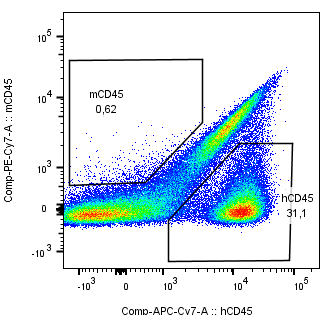

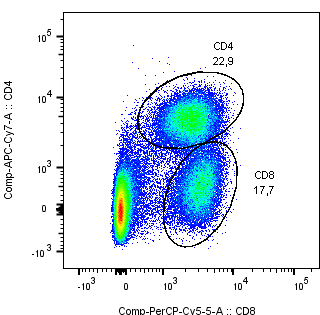

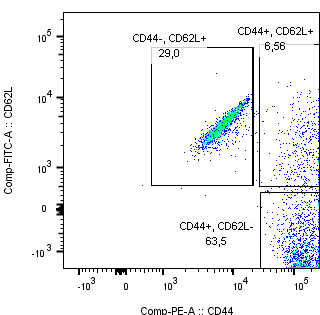

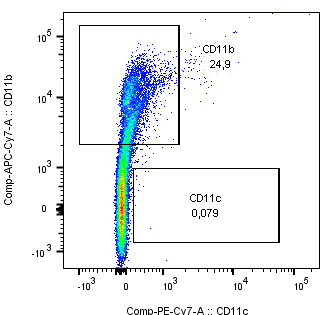

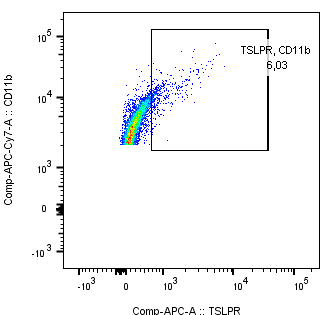

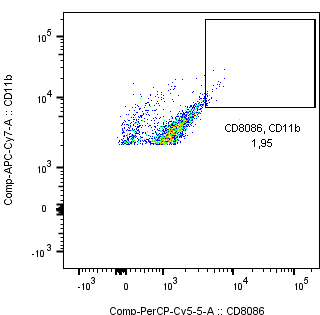

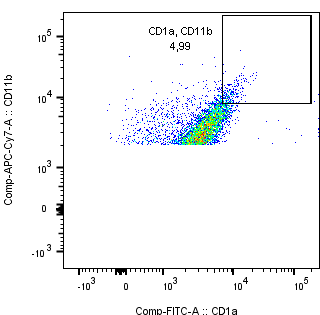

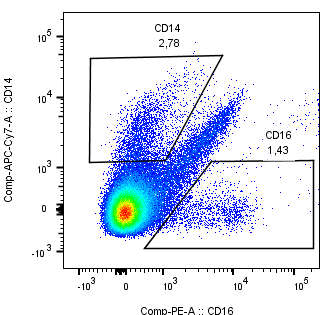

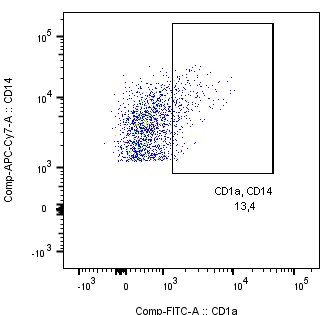

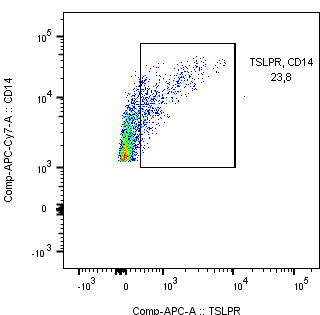

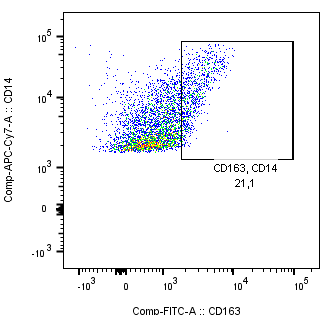

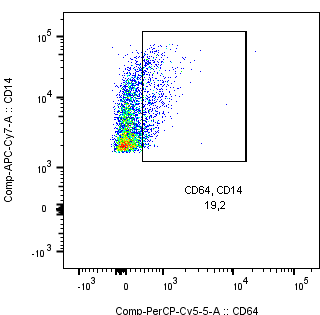

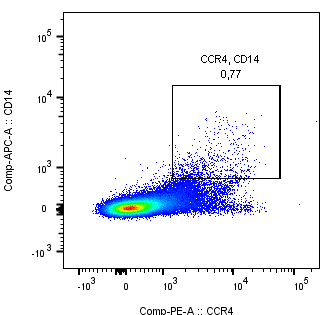

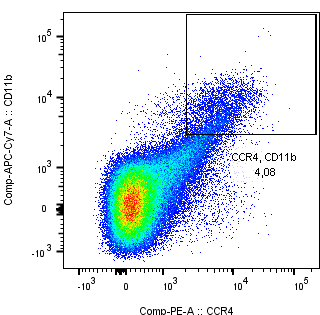

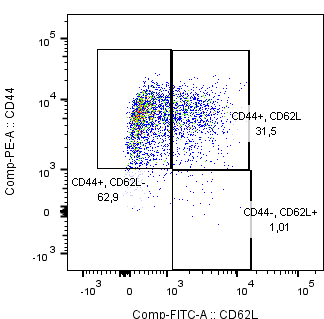


**mCD45+ hCD45+**

**CD4+ CD44± CD62L±**

**CD4+ CD8+**

**CD11b+ CD11c+**

**CD11b+ TSLPR+**

**CD11b+ CD80/86+**

**CD11b+ CD1a**

**CD14+ CD1a+**

**CD14+ TSLPR+**

**CD14+ CD64+**

**CD14+ CD163+**

**CD11b+ CCR4+**

**CD14+ CCR4+**

**CD8+ CD44± CD62L±**

**Fig. S1 Gating strategy for human leukocytes isolated from mouse spleen.**

**Table S1. Cellular markers used to define immune cells**

| **Marker** | **Definition** |
| --- | --- |
| CD19+ CD27+ IgD+ | Unswitched memory B-cell |
| CD19+ CD27+ IgD- | Switched memory B-cell |
| CD19+ CD38+ | Plasma cell |
|  |  |
| CD4+ CD45RO+ CD62L- CCR7- | Effector memory CD4+ T- cell |
| CD4+ CD45RO+ CD62L+, CCR7+ | Central memory |
| CD4+ CD103+ | Mucosal regulatory CD4+ T cell |
| CD4+ CCR4+ | Th2, effector regulatory T- cell |
| CD4+ CD25+ CD127- | Regulatory T-cell |
|  |  |
| Th1 | CD4+ CXCR3+ |
| Th2 | CD4+ CCR4+ CCR6- |
| Th17 | CD4+ CCR4+ CCR6+ |
| Th22 | CD4+ CCR4+ CCR6+ CCR10+ |
|  |  |
| CD4+ CD134+ | Activated CD4+ T cell |
| CD4+ CD69+ | Activated CD4+ T cell |
| CD4+ CD25+ | Activated CD4+ T cell |
|  |  |
| CD14+ | MC* |
| CD14+ TSLPR+ | MC, expressing TSLPR |
| CD14+ CD64+ | M1MC |
| CD14+ CD163+ CD206+ | M2 MC, scavenging cells |
| CD14+ CD1a | MC CD1a expressing |
|  |  |
| CD11b+ | cDC1** |
| CD11b+ CD80/86+ | cDC1, mature |
| CD11b+ HLADR+ | cDC1, presenting |
| CD11b+ TSLPR+ | cDC1 TSLPR expressing |
| CD11b+ CD1a+ | cDC1 CD1a expressing |

*Monocyte

******Conventional dendritic cell

TSLPR: Thymic stromal lymphopoietin receptor

**Table S2. Monoclonal antibodies used in labelling of surface markers of leucocytes**

| **Surfacemarker (anti human)** | **Colour** | **Clone** |
| --- | --- | --- |
| CD19 | Peridine-chlorophyll-protein complex cyanine dye (PerCP-Cy^TM^ 5.5) | HIB19 |
| CD38 | Phycoerythrin (PE) | HB-7 |
| CD27 | Pe-Cy7 | LG.3A10 |
| IgD | Fluorescin isothiocyanate (FITC) | LA6-2 |
| CD4 | Allophycocyanin (APC)- Cy7 | OKT4 |
| CD44 | PE | BJ18 |
| CD62L | FITC | DREG-56 |
| CD8 | PerCP-Cy^TM^ 5.5 | HIT8a |
| CD103 | APC | Ber-ACT8 |
| CD25 | PE-Cy7 | BC96 |
| CD294 (CRTH2) | APC | BM16 |
| CD14 | APC-Cy7 | HCD14 |
| CCR2 | PE-Cy7 | K036C2 |
| CD80/86 | PerCP-Cy^TM^ 5.5 | IT2.2 |
| TSLPR | APC | 1B4 |
| CD1a (biotin)/secondary Ab streptavidin | FITC | HI149 |
| CD64 | PerCP-Cy^TM^ 5.5 | 10.1 |
| CD163 | FITC | GHI/61 |
| CD16 | PE | 3G8 |
| CD11b | APC-Cy7 | M1/70 |
| CD11c | PE-Cy7 | 3.9 |
| CD3 | APC-Cy7 | HIT3a |
| CD56 | FITC | HCD56 |
| CD94 | PE | DX22 |
| KIR | PE-Cy7 | DX27 |
| CD127 | PerCP-Cy^TM^ 5.5 | A019D5 |

**Table S3 Data set of variables changed upon challenge with ethanol in the NSG-UC mouse model**

|  | **Mean** | | **SD** | | **IQR** | | **n** | | **Δ** | **p-value** | **95% CI** | |
| --- | --- | --- | --- | --- | --- | --- | --- | --- | --- | --- | --- | --- |
| **Leukocytes [% FoP]** | **Control** | **Challenge** | **Control** | **Challenge** | **Control** | **Challenge** | **Control** | **Challenge** |  |  | **lower** | **upper** |
| CD11b+ CD1a | 6,22 | 12,62 | 3,31 | 6,4 | 3,91 | 8,51 | 20 | 20 | 6,4 | 5,00E-04 | -9,63 | -3,03 |
| CD11b+ CD86+ | 28,73 | 28,94 | 27,12 | 23,29 | 49,9 | 47,9 | 32 | 36 | 0,21 |  |  |  |
| CD14+ CD1a | 13,76 | 31,48 | 12,3 | 21,4 | 13,92 | 25,82 | 31 | 36 | 17,72 | 9E-05 | -26,03 | -9,23 |
| CD14+ CD64+ | 5,75 | 15,56 | 4,9 | 10,65 | 7,87 | 15,03 | 32 | 36 | 9,81 | 8E-06 | -13,78 | -5,84 |
| CD14+ CD163+ | 10,55 | 23,59 | 8,62 | 17,06 | 9,55 | 16 | 32 | 36 | 13,04 | 1E-04 | -19,51 | -6,56 |
| CD14+ CD206+ | 1,02 | 5,95 | 0,85 | 5,5 | 1,61 | 7,31 | 10 | 12 | 4,93 | 1E-02 | -8,45 | -1,4 |
| CD14+ CD163+ CD206+ | 4,07 | 11,52 | 3,94 | 16,12 | 4,58 | 8,85 | 24 | 28 | 7,45 | 0,02 | -13,89 | -1,02 |
| CD14+ TSLPR+ | 1,49 | 5,12 | 2,78 | 12,69 | 0,86 | 2,05 | 35 | 39 | 3,63 | 0,08 | -7,8 | 0,57 |
|  |  |  |  |  |  |  |  |  |  |  |  |  |
|  |  |  |  |  |  |  |  |  |  |  |  |  |
| Score |  |  |  |  |  |  |  |  |  |  |  |  |
| Clinical Score | 1,06 | 7,78 | 1,13 | 7,72 | 2 | 9 | 32 | 38 | 6,72 | 4,90E-06 | -9,29 | -4,15 |
| Histological Score | 0,96 | 4,4 | 0,91 | 4,4 | 1,5 | 4 | 31 | 37 | 3,44 | 4E-09 | -4,39 | -2,48 |

**Table S4 Data set of variables changed upon challenge with ethanol and treated with infliximab.**

|  | **Mean** | | | **SD** | | | **IQR** | | | **n** | | |
| --- | --- | --- | --- | --- | --- | --- | --- | --- | --- | --- | --- | --- |
| **Leukocytes [% FoP]** | **Control** | **Challenge** | **Infliximab** | **Control** | **Challenge** | **Infliximab** | **Control** | **Challenge** | **Infliximab** | **Control** | **Challenge** | **Infliximab** |
| CD11b+ TSLPR+ | 1,48 | 1,57 | 1,39 | 0,91 | 1,71 | 0,98 | 0,94 | 1,75 | 1,52 | 8 | 8 | 8 |
| CD11b+ CD1a+ | 6,84 | 15,17 | 10,88 | 4,12 | 7,91 | 7,28 | 6,85 | 10,82 | 12,94 | 8 | 8 | 8 |
| CD11b+ CD80/86+ | 4,27 | 5,64 | 7,33 | 2,61 | 3,94 | 6,24 | 3,94 | 4,07 | 8,56 | 8 | 8 | 8 |
| CD14+ TSLPR+ | 2,2 | 15,85 | 0,44 | 3,55 | 24,31 | 0,64 | 2,27 | 20,96 | 0,27 | 7 | 8 | 8 |
| CD14+ CD1a+ | 10,79 | 39,33 | 12,12 | 7 | 31,25 | 3,73 | 10,19 | 34,32 | 4,13 | 7 | 8 | 8 |
| CD14+ CD64+ | 6,45 | 23,51 | 16,35 | 2,33 | 5,32 | 3,38 | 2,85 | 3,7 | 3,12 | 8 | 8 | 8 |
| CD14+ CD163+ | 10,77 | 27,78 | 9,69 | 8,99 | 23,06 | 7,34 | 11,74 | 17,55 | 12,01 | 8 | 8 | 8 |
| CD4+ CCR4+ | 6,26 | 11,69 | 6,07 | 5,99 | 7,46 | 3,88 | 7,65 | 11,7 | 5,79 | 5 | 8 | 8 |
|  |  |  |  |  |  |  |  |  |  |  |  |  |
| **Score** |  |  |  |  |  |  |  |  |  |  |  |  |
| Clinical Score | 1,25 | 11,65 | 5,25 | 1,16 | 10,19 | 4,46 | 2 | 10,25 | 3 | 8 | 8 | 7 |
| Histological Score | 0,87 | 7 | 0,62 | 0,83 | 2,5 | 1 | 1,25 | 3,5 | 1 | 8 | 8 | 8 |
|  |  |  |  |  |  |  |  |  |  |  |  |  |
| **mRNA [lg-delta CT]** |  |  |  |  |  |  |  |  |  |  |  |  |
| mTGFß1 | 0,07 | 0,10 | 0,10 | 0,02 | 0,04 | 0,03 | 0,03 | 0,03 | 0,03 | 8 | 8 | 8 |
| HGF | 0,17 | 0,28 | 0,19 | 0,07 | 0,13 | 0,08 | 0,10 | 0,20 | 0,10 | 8 | 8 | 8 |
| mTARC | 0,04 | 0,12 | 0,07 | 0,02 | 0,05 | 0,01 | 0,02 | 0,07 | 0,02 | 8 | 8 | 7 |
| hIFNγ | 0,01 | 3,68 | 0,05 | 0,02 | 7,37 | 0,05 | 0,01 | 3,68 | 0,05 | 4 | 4 | 4 |

|  | **Δ** |  | **95% CI** | | **Δ** |  | **95% CI** | |
| --- | --- | --- | --- | --- | --- | --- | --- | --- |
| **Leukocytes [% FoP]** | **Challenge / Control** | **p-value** | **lower** | **upper** | **Infliximab / Challenge** | **p-value** | **lower** | **upper** |
| CD11b+ TSLPR+ | 0,09 |  |  |  | -0,18 |  |  |  |
| CD11b+ CD1a+ | 8,33 | 0,05 | -0,05 | 16,7 | -4,29 |  |  |  |
| CD11b+ CD80/86+ | 1,37 |  |  |  | 1,69 |  |  |  |
| CD14+ TSLPR+ | 13,65 |  |  |  | -15,41 |  |  |  |
| CD14+ CD1a+ | 28,54 | 0,02 | 3,64 | 53,43 | -27,21 | 0,02 | -51,27 | -3,16 |
| CD14+ CD64+ | 17,06 | 1E-07 | 12,16 | 21,95 | -7,16 | 0.0016 | -12,05 | -2,26 |
| CD14+ CD163+ | 17,01 |  |  |  | -18,09 |  |  |  |
| CD4+ CCR4+ | 5,43 |  |  |  | -5,62 |  |  |  |
|  |  |  |  |  |  |  |  |  |
| **Score** |  |  |  |  |  |  |  |  |
| Clinical Score | 10,4 | 0,004 | 1,2 | 6,8 | -6,4 |  |  |  |
| Histological Score | 6,13 | 1E-06 | 2,19 | 5,07 | -6,38 | 0,01 | -2,85 | -5,04 |
|  |  |  |  |  |  |  |  |  |
| **mRNA [lg-delta CT]** |  |  |  |  |  |  |  |  |
| mTGFß1 | 0,03 | 0,08 |  |  | -0,01 |  |  |  |
| HGF | 0,12 | 0,05 | -2,2 | 0,23 | -0,10 |  |  |  |
| mTARC | 0,08 | 1E-05 | -2,3 | -1 | -0,05 | 0,005 | -0.09 | -0.01 |
| hIFNγ | 3,67 |  |  |  |  |  |  |  |

**Table S5 Data set of variables changed upon challenge with ethanol and treated with pitrakinra.**

|  | **Mean** | | | **SD** | | | **IQR** | | | **n** | | |
| --- | --- | --- | --- | --- | --- | --- | --- | --- | --- | --- | --- | --- |
| **Leukocytes [% FoP]** | **Control** | **Challenge** | **Pitrakinra** | **Control** | **Challenge** | **Pitrakinra** | **Control** | **Challenge** | **Pitrakinra** | **Control** | **Challenge** | **Pitrakinra** |
| CD11b+ TSLPR+ | 2,8 | 4,36 | 7,41 | 4,26 | 6,51 | 10,39 | 5,15 | 10,3 | 15,18 | 12 | 12 | 11 |
| CD11b+ CD1a+ | 5,91 | 10,91 | 12,99 | 2,78 | 4,8 | 2,71 | 3,57 | 9,12 | 2,8 | 12 | 12 | 11 |
| CD11b+ CD80/86+ | 13,72 | 12,29 | 8,53 | 14,96 | 9,54 | 8,52 | 27,58 | 18,16 | 14,78 | 12 | 12 | 11 |
| CD14+ TSLPR+ | 0,53 | 1,33 | 0,41 | 0,41 | 1,09 | 0,6 | 0,64 | 2,08 | 0,61 | 12 | 12 | 11 |
| CD14+ CD1a+ | 5,31 | 14,24 | 8,27 | 2,59 | 6,89 | 3,83 | 3,49 | 8,99 | 4,52 | 12 | 12 | 11 |
| CD14+ CD64+ | 1,5 | 12,51 | 3,14 | 2,59 | 14,44 | 5 | 1,78 | 15,98 | 4,3 | 12 | 12 | 11 |
| CD14+ CD163+ | 5,59 | 23,41 | 8,65 | 4,01 | 17,99 | 6,73 | 6,36 | 10,72 | 12,3 | 12 | 12 | 11 |
|  |  |  |  |  |  |  |  |  |  |  |  |  |
| **Score** |  |  |  |  |  |  |  |  |  |  |  |  |
| Clinical Score | 0,83 | 4,83 | 4,16 | 1,11 | 4,21 | 3,09 | 2 | 5,5 | 3 | 12 | 12 | 12 |
| Histological Score | 0,51 | 1 | 2 | 0,99 | 1,16 | 2,23 | 1 | 2 | 2 | 12 | 12 | 11 |
|  |  |  |  |  |  |  |  |  |  |  |  |  |
| **mRNA [lg-delta CT]** |  |  |  |  |  |  |  |  |  |  |  |  |
| mTGFß1 | 0,06 | 0,11 | 0,07 | 0,02 | 0,05 | 0,05 | 0,02 | 0,07 | 0,08 | 8 | 8 | 7 |
| HGF | 0,17 | 0,39 | 0,12 | 0,15 | 0,33 | 0,11 | 0,17 | 0,46 | 0,10 | 8 | 8 | 6 |
| mTARC | 0,03 | 0,05 | 0,06 | 0,02 | 0,05 | 0,02 | 0,02 | 0,05 | 0,02 | 7 | 6 | 7 |
| hIFNγ | 0 | 0,002 | 0,2 | 0 | 0 | 0,22 | 0 | 0 | 0,36 | 8 | 8 | 6 |
| hTNFα | 0 | 0 | 0,12 | 0 | 0 | 0,15 | 0 | 0 | 0,03 | 8 | 8 | 7 |

|  | **Δ** |  | **95% CI** | | **Δ** |  | **95% CI** | |
| --- | --- | --- | --- | --- | --- | --- | --- | --- |
| **Leukocytes [% FoP]** | **Challenge / Control** | **p-value** | **lower** | **upper** | **Pitrakinra / Challenge** | **p-value** | **lower** | **upper** |
| CD11b+ TSLPR+ | 1,56 |  |  |  | 3,05 |  |  |  |
| CD11b+ CD1a+ | 5 | 0,004 | 1,39 | 8,6 | 2,08 |  |  |  |
| CD11b+ CD80/86+ | -1,43 |  |  |  | -3,76 |  |  |  |
| CD14+ TSLPR+ | 0,8 | 0,03 | 0,03 | 1,57 | -0,92 | 0,01 | -1,71 | -0,13 |
| CD14+ CD1a+ | 8,93 | 0,0002 | 4,09 | 13,76 | -5,97 | 0,01 | -10,9 | -1,02 |
| CD14+ CD64+ | 11,01 | 0,01 | 1,93 | 20,08 | -9,37 | 0,04 | -18,6 | -0,08 |
| CD14+ CD163+ | 17,82 | 0,001 | 6,32 | 29,3 | -14,76 | 0,01 | -26,5 | -3,01 |
|  |  |  |  |  |  |  |  |  |
| **Score** |  |  |  |  |  |  |  |  |
| Clinical Score | 4 | 0.003 |  |  | -0,67 |  |  |  |
| Histological Score | 0,49 | 2E-06 |  |  | 1 |  |  |  |
|  |  |  |  |  |  |  |  |  |
| **mRNA [lg-delta CT]** |  |  |  |  |  |  |  |  |
| mTGFß1 | 0,05 | 0,05 | 0,00 | 0,10 | -0,04 |  |  |  |
| HGF | 0,22 | 0,06 |  |  | -0,27 |  |  |  |
| mTARC | 0,02 | 0,02 | 0 | 0,1 | 0,01 |  |  |  |
| hIFNγ | 0,00 |  |  |  | 0,20 | 1E-02 | 0,04 | 0,36 |
| hTNFα | 0,00 |  |  |  | 0,12 | 0,01 | 0,04 | 0,36 |
